# Urotensin II Induces Interleukin 8 Expression in Human Umbilical Vein Endothelial Cells

**DOI:** 10.1371/journal.pone.0090278

**Published:** 2014-02-21

**Authors:** Chung-Yi Lee, Yi-Tin Tsai, Shih-Hurng Loh, Ju-Chi Liu, Tso-Hsiao Chen, Hung-Hsing Chao, Tzu-Hurng Cheng, Jin-Jer Chen

**Affiliations:** 1 Department of Cardiovascular Surgery, Tri-Service General Hospital, Taipei, Taiwan, Republic of China; 2 Department of Pharmacology, National Defense Medical Center, Taipei, Taiwan, Republic of China; 3 Department of Medicine, Taipei Medical University, Taipei, Taiwan, Republic of China; 4 Division of Nephrology, Department of Internal Medicine, Wan Fang Hospital, Taipei Medical University, Taipei, Taiwan, Republic of China; 5 Shin Kong Wu Ho-Su Memorial Hospital, Taipei, Taiwan, Republic of China; 6 Department of Biochemistry, School of Medicine, China Medical University, Taichung, Taiwan, Republic of China; 7 Graduate Institute of Clinical Medicine, College of Medicine, China Medical University, Taichung, Taiwan, Republic of China; 8 Institute of Biomedical Sciences, Academia Sinica, Taipei, Taiwan, Republic of China; Institute of Biomedical Sciences, Taiwan

## Abstract

**Background:**

Urotensin II (U-II), an 11-amino acid peptide, exerts a wide range of actions in cardiovascular systems. Interleukin-8 (IL-8) is secreted by endothelial cells, thereby enhancing endothelial cell survival, proliferation, and angiogenesis. However, the interrelationship between U-II and IL-8 as well as the detailed intracellular mechanism of U-II in vascular endothelial cells remain unclear. The aim of this study was to investigate the effect of U-II on IL-8 expression and to explore its intracellular mechanism in human umbilical vein endothelial cells.

**Methods/Principal Findings:**

Primary human umbilical vein endothelial cells were used. Expression of IL-8 was determined by real-time quantitative polymerase chain reaction, enzyme-linked immunosorbent assay, and luciferase reporter assay. Western blot analyses and experiments with specific inhibitors were performed to reveal the downstream signaling pathways as concerned. U-II increased the mRNA/protein levels of IL-8 in human umbilical vein endothelial cells. The U-II effects were significantly inhibited by its receptor antagonist [Orn^5^]-URP. Western blot analyses and experiments with specific inhibitors indicated the involvement of phosphorylation of p38 mitogen-activated protein kinase and extracellular signal-regulated kinase in U-II-induced IL-8 expression. Luciferase reporter assay further revealed that U-II induces the transcriptional activity of IL-8. The site-directed mutagenesis indicated that the mutation of AP-1 and NF-*k*B binding sites reduced U-II-increased IL-8 promoter activities. Proliferation of human umbilical vein endothelial cells induced by U-II could be inhibited significantly by IL-8 RNA interference.

**Conclusion/Significance:**

The results show that U-II induces IL-8 expression in human umbilical vein endothelial cells via p38 mitogen-activated protein kinase and extracellular signal-regulated kinase signaling pathways and IL-8 is involved in the U-II-induced proliferation of human umbilical vein endothelial cells.

## Introduction

The vascular endothelium plays important roles in the control of vascular tone, platelet activity, leukocyte adhesion, and angiogenesis by producing several regulatory factors [1]. Urotensin II (U-II), an 11 amino acid cyclic peptide, is generally agreed to be the most potent endogenous vasoconstrictor discovered to date [2]. The human form of U-II is synthesized by proteolytic cleavage of a precursor, the prepro-U-II. Prepro-U-II mRNA was found by dot-blot analysis in many tissues, such as spinal cord, kidney, spleen, small intestine, thymus, prostate, pituitary gland and adrenal gland and, to a lesser extent, in stomach, pancreas, ovary, and liver [3], [4]. Both U-II and its receptor GPR 14 (UT receptor, UT) are expressed in many tissues, including the endothelium [5]. In recent years, knowledge concerning the impact of U-II on endothelial cell function has increased. Evidence supports the involvement of U-II in endothelial cell proliferation [6] and angiogenesis [7], [8]. U-II and its receptor are well expressed in cultured endothelial cells, and when tested in Matrigel assays, U-II exerted a significant pro-angiogenic activity [8]. Cytokines, such as interleukin-8 (IL-8), have been recognized as angiogenic factors. Secretion of IL-8 from cancer cells can aggravate the proliferation and survival of cancer cells, in part by autocrine signaling pathways [9]. In addition, tumor-derived IL-8 can activate endothelial cells to promote angiogenesis. IL-8 is secreted not only by tumor cells, but also by endothelial cells, thereby enhancing endothelial cell survival, proliferation, and angiogenesis [9]. Several signaling pathways have been implicated in the induction of IL-8 expression, including nuclear factor-*κ*B (NF-*κ*B)- and activating protein (AP)-1-dependent pathway which are activated by mitogen-activated protein kinases (MAPKs) pathway, including extracellular signal-regulated kinase (ERK), c-Jun N-terminal kinase (JNK) and p38 MAPK pathway in endothelial cells [10], [11]. In addition, U-II has been shown to activate MAPK signal transduction pathways in different cell types [12]–[14]. Although U-II has been found to induce the proliferation of endothelial cells [6], its induction of IL-8 gene expression in vascular endothelial cells has not been studied, and the crosstalk between U-II and IL-8 in activation of endothelial cells remains unknown. This study aimed to define the molecular mechanisms of IL-8 gene expression in human umbilical vein endothelial cells (HUVECs) stimulated with U-II, and to explore the involvement of IL-8 in the proliferation of endothelial cells induced by U-II. Results show that U-II induced IL-8 expression which was partly reduced by mitogen-activated protein kinase (MAPK) inhibitors, and IL-8 RNA interference inhibited U-II-induced proliferation of human umbilical vein endothelial cells.

## Materials and Methods

### Materials

[Orn^5^]-URP, an U-II receptor pure antagonist, was obtained from Tocris Bioscience (Bristol, UK). Fetal bovine serum was from Hyclone (Logan, UT, USA). Dulbecco's modified Eagle's medium (DMEM) was from GiBco (Invitrogen-Gibco, Carlsbad, CA, USA). U-II, U0126, SB203580, and all other chemicals of reagent grade were obtained from Sigma-Aldrich (St. Louis, MO, USA).

### Endothelial cell culture

Human umbilical vein endothelial cells (HUVECs) were obtained from PromoCell (Heidelberg, Germany) as cryopreserved cells [15]. After thawing, cells were plated in cultured flasks and cultured to confluence in MCBD 131 medium (PromoCell) containing 28 mM hydroxyethylpiperazine ethanesulfonic acid, 2% fetal calf serum, 0.1 ng/ml human recombinant epidermal growth factor, 1 ng/ml human recombinant basic fibroblast growth factor, 50 µg/ml gentamycin, 50 ng/ml amphotericin B, and 1 µg/ml synthetic hydrocortisone and supplemented with a mixture (PromoCell) containing endothelial cell growth factor and heparin. Cells were grown at 37°C in a humidified 5% CO_2_ atmosphere for 3∼4 days. Confluent cultures between passages 2–3 were used in this study to minimize age-dependent variation in the level of apoptosis.

### Determination of IL-8

IL-8 levels in cell medium were determined by ELISA. Commercially available ELISA kits for the quantification of IL-8 (R & D Systems, Minneapolis, MN, U.S.A.) were used as according to the manufacturer's protocol.

### RNA extraction and quantitative polymerase chain reaction (qPCR) analysis

Total RNA was extracted from HUVECs using the TRIzol method according to the protocol recommended by the manufacturer (Invitrogen, Carlsbad, CA, USA), and used to synthesize single-stranded complementary (c)DNA with a High-Capacity cDNA Reverse Transcription Kit (Applied Biosystems, Foster City, CA, USA) as described in a previous report [16]. IL-8 messenger (m)RNAs were quantified using TaqMan Gene Expression Master Mix (Applied Biosystems) with specific primers in an ABI 7300 Real-Time PCR System (Applied Biosystems). TaqMan Gene Expression Assay kits containing specific primers for IL-8 (lot no. Hs00174103_m1), and GAPDH (lot no. Hs99999905_m1) were obtained from Applied Biosystems. Specific primers for GAPDH were used to normalize the amount of sample added. Relative amounts of IL-8 mRNA were quantitated using the comparative Ct method. All quantifications were performed on triplicate samples of three separate experiments.

### Immunoblotting analysis

Cells were rinsed twice with cold phosphate buffered saline and incubated in lysis buffer (50 mM Tris–Cl, pH 7.4 containing 150 mM NaCl, 1 mM EDTA, 1% Triton X-100, 1 mM sodium vanadate, 2.5 mM sodium pyrophosphate, 2 µg/ml leupeptin and aprotinin, 1 mM PMSF and 15% glycerol) for 15 min on ice. The cell lysate was cleared by centrifugation at 14,000 rpm for 10 min and the supernatant was used for Western immunoblotting analysis. Equal amounts of cellular protein (10 µg) were separated by SDS-PAGE followed by electrotransfer to PVDF membranes. Membranes were successively incubated with rabbit polyclonal antibodies against phosphorylated and total p38, p44/42 and JNK MAPKs (Cell Signaling, Beverly, MA, USA) at 1∶1000 dilution overnight at 4°C followed by goat anti-rabbit alkaline phosphatase conjugated-secondary antibody (Cell Signaling) at 1∶2000 dilution for 1 h at room temperature. Protein bands were visualized by the Enhanced Chemifluorescence (ECF) detection method (Amersham Pharmacia Biotech, Piscataway, NJ, USA) according to the manufacturer's instructions. Membranes were scanned with a fluorescence scanner for visualization of protein bands and the intensity of the bands was quantified using Quantity One Image Acquisition and Analysis Software (Bio-Rad).

### Luciferase Reporter and Electrophoretic Mobility Shift Assay

The constructions of the IL-8 proximal promoter, the site-directed mutagenesis of the IL-8 AP-1, nuclear factor (NF)-IL-6, and NF-*κ*B sites [17], all kindly provided by Dr. Jeremy JW Chen (Institute of Biomedical Sciences, National Chung-Hsing University, Taichung, Taiwan), were used for luciferase reporter assay. Briefly, 5×10^4^ HUVECs were cotransfected with the individual IL-8 promoter construct and a pSV-β-galactosidase plasmid (Promega) with LipofectAMINE (Invitrogen), and the cells were treated with U-II at the designated concentrations for 24 h. The cell lysate was harvested and the genes were identified by luciferase activity using the Dual-Light Luciferase Reporter Gene Assay System (Invitrogen). All experiments, including those on nontransfected and vector-treated cells (as negative controls), were done in triplicate.

Double-stranded oligonucleotides containing two sequences coding the IL-8-specific AP-1 (5′-AAAAGTGTGA*TGACTCA*GGTTTGTGA*TGACTCA*GGTTTG-3′ annealed with 5′-AAACAAACC*TGAGTCA*TCACCC*TGAGTCA*TCACACT-3′) and NF-*κ*B (5′-AAAAAAAATCG*TGGAATTTCC*CCCGAATCG*TGGAATTTCC*CCCGA-3′ annealed with 5′-AAATCGGG*GGAAATTCCA*CGATTCGGG*GGAAATTCCA*CGATT-3′) binding sites (italicized) were designed for electrophoretic mobility shift assay [18]. Oligonucleotides were labeled with digoxigenin-11-dUTP (Roche Molecular Biochemicals) using Klenow polymerase (Invitrogen) and purified by MicroSpin G-50 columns (Amersham Pharmacia). The details of nuclear extract preparation, electrophoresis, and detection have been described previously [17], [19].

### Knock down of IL-8 in HUVECs by siRNA and cell proliferation assay

HUVECs were seeded at a density of 2×10^5^ cells/well in 2 ml culture medium in 6-well tissue culture plates. After overnight incubation, the cells were transfected with IL-8 siRNA (sc-39631) or control siRNA (sc-37007, Santa Cruz Biotechnology, Inc.) using siRNA transfection reagent according to the manufacturer's instruction (Santa Cruz Biotechnology Inc). After transfection, the cells were split, and incubated for 24 h with DMEM containing 10% FBS for Qpcr, ELISA, or proliferation assays. Proliferation was assessed by counting and 5-bromo-2′-deoxyuridine (BrdU) labeling of cells as previously described [20]. Data are presented as the mean±S.E.M. of 9–12 determinations in three to four different cell preparations and normalized to the untreated sample×100 (*i.e*. percentage of control).

### Statistics

Results are expressed as mean±S.E.M. The number of experiments (*n*) was indicated in figure legends. Statistical analysis was performed using Student's *t*-test or analysis of variance (ANOVA) followed by a Dunnett multiple comparison test using GraphPad Prism (GraphPad Software, San Diego, CA, USA). A value of *P*<0.05 was considered to be statistically significant.

## Results

### U-II induces IL-8 expression in HUVECs

**Figure 1:**
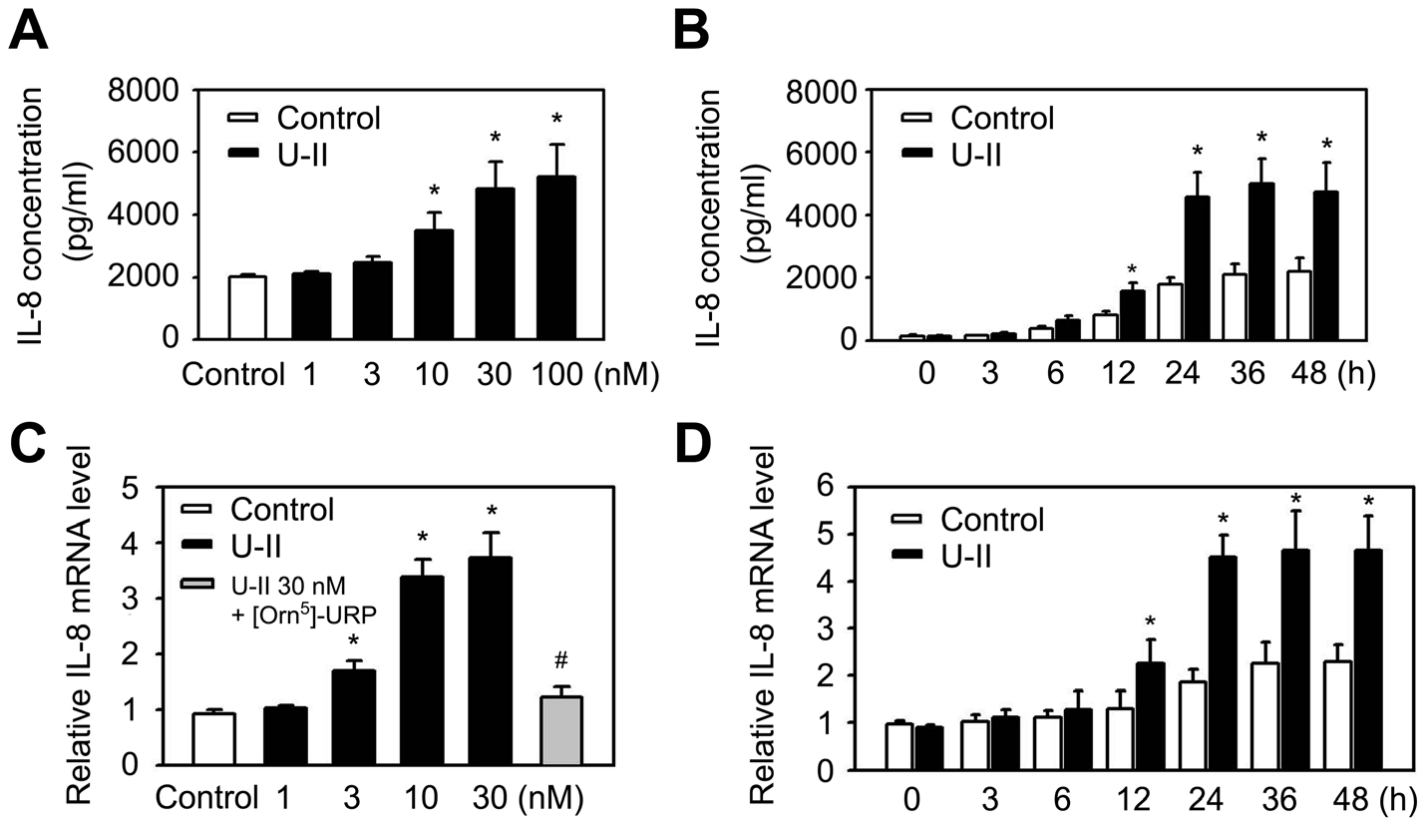
Effects of U-II on the IL-8 expression in HUVECs. (A, B) Effects of U-II on the IL-8 protein expression in supernatants of HUVECs. IL-8 protein content was assayed in culture supernatants by IL-8 ELISA (R&D Systems) according to the manufacturer's instructions. A, HUVECs were left untreated (Control) or were treated with U-II for 24 h at the indicated concentrations. B, HUVECs were left untreated (Control) or were treated with U-II (30 nM) for the indicated times. (C, D) IL-8 mRNA expression is induced by U-II. Total mRNA extracted from HUVECs that were treated with vehicle or U-II (24 h) for varying concentrations (C) or varying times (D) as shown. The mRNA quantity of IL-8 in each sample was detected by a qPCR with specific primers for the IL-8. Cells were preincubated with [Orn^5^]-URP (1 µM) for 1 h before their incubation with U-II (30 nM) for 24 h. Results were shown as the mean±S.E.M. (*n* = 6). *P<0.05 vs. control (Cont); ^#^ *P*<0.05 vs. U-II alone.

To evaluate the influence of U-II on IL-8 expression in endothelial cells, ELISA analyses were performed. Interestingly, these experiments demonstrated a time- and concentration-dependent induction of IL-8 expression in HUVECs treated with U-II (Fig. 1A, B). To determine whether U-II plays a role in the IL-8 mRNA expression, IL-8 mRNA expression in cells treated with U-II was measured. Consistent with previously reported protein expression data, application of U-II induced IL-8 mRNA expression in a concentration- and time-dependent manner (Fig. 1. C, D). IL-8 mRNA was induced rapidly by U-II to the maximum level at 1 h of incubation. Elevated IL-8 mRNA levels in U-II-treated cells were sustained for extended periods of time. The levels of GAPDH mRNA were unaffected by U-II. In the presence of [Orn^5^]-URP (an antagonist to the U-II receptor; 1 µM), the enhancement effects of U-II on IL-8 mRNA expression were completely abolished (Fig. 1D).These data show that U-II induces IL-8 gene expression in HUVECs.

### Involvement of a signaling pathway in the induction of IL-8 following U-II treatment

**Figure 2:**
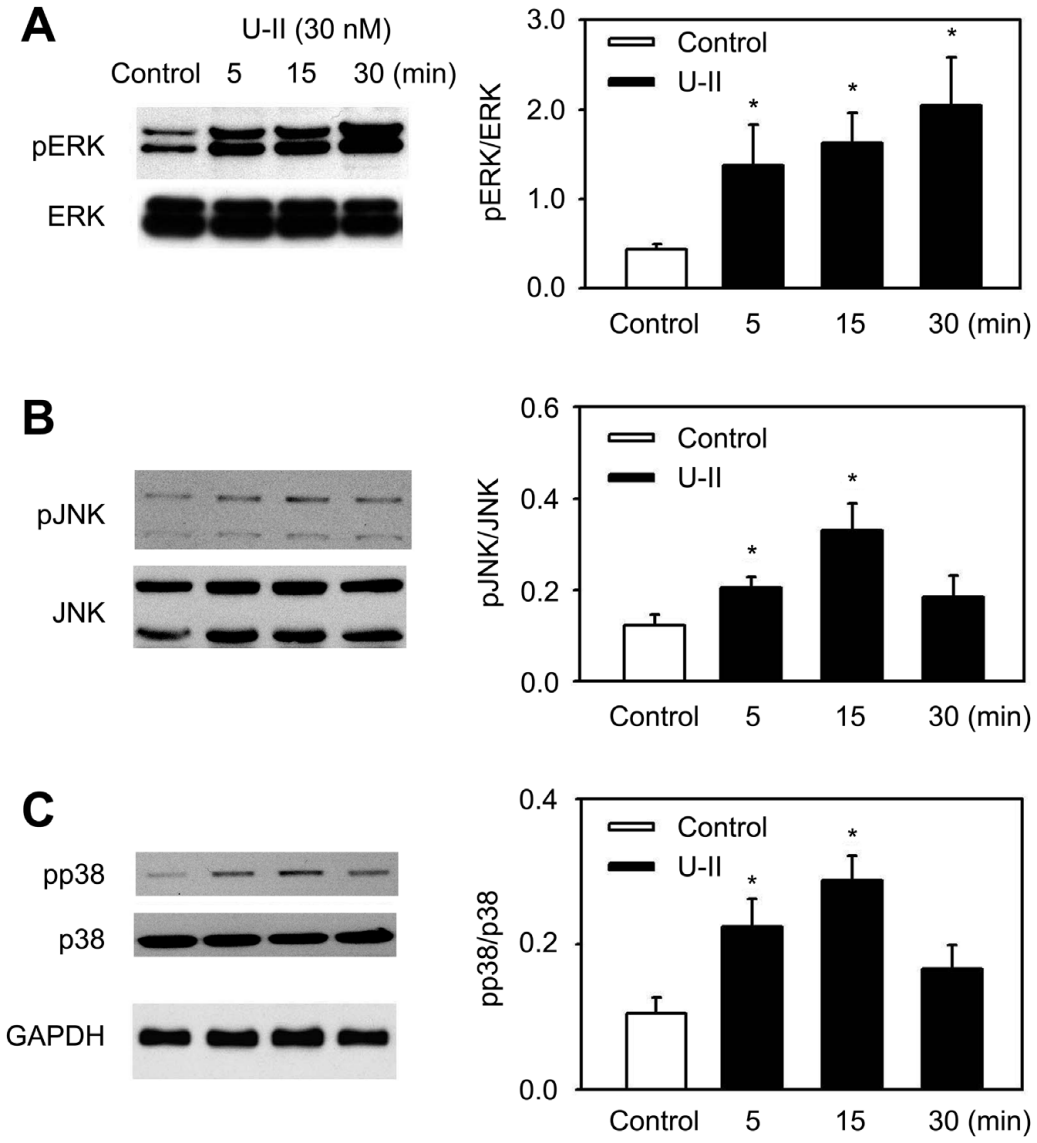
Effects of U-II on MAPK activation. HUVECs were serum-starved for 24 h and then treated with U-II (30 nM) for the indicated periods of time. The levels of phosphorylated and total ERK (A), p38 (B), and JNK (C) MAP kinases were analyzed by Western immunoblotting. Results were shown as the mean±S.E.M. (*n* = 4). *, *p*<0.05 was considered significant.

**Figure 3:**
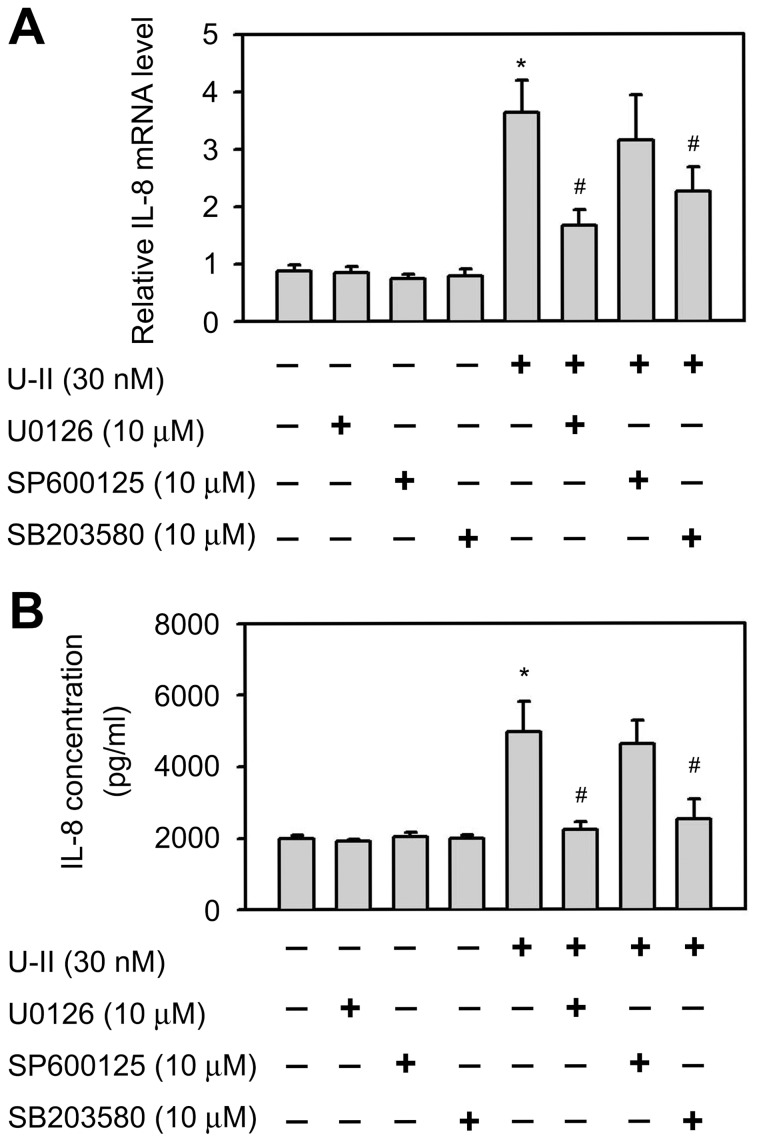
Identification of MAPK pathway involvement in IL-8 gene activation in response to U-II stimulation. A. Effects of pharmacological inhibitors of MAPK activation on U-II induction of IL-8 mRNA levels. IL-8, and GAPDH mRNA levels were determined by qPCR with specific primers for the IL-8 and GAPDH. B. Effects of pharmacological inhibitors of MAPK activation on U-II induction of IL-8 protein secretion. HUVECs were first incubated in medium ± inhibitor (U0126, 10 µM; SB 203850, 10 µM; SP600125, 10 µM) for 1 h and then treated with U-II (30 nM) for an additional 24 h. Data shown are as the mean ± SEM. (*n* = 6). *, *P*<0.05 versus control. #, *P*<0.05 versus U-II alone.

U-II is known to activate MAPK signaling pathways in a variety of cells [8], [12], [20], [21]. The involvement of MAPK signaling pathways in the U-II induction of IL-8 mRNA expression was explored. U-II stimulated the phosphorylation of ERK, p38 and JNK MAPKs in a time-dependent manner in HUVECs; enhanced phosphorylation was observed as early as 10 min after exposure to U-II and leveled off to control levels after 1 h (Figure 2). These data indicated that MAPK signaling pathways may be involved in the U-II induction of IL-8 mRNA expression. To determine the involvement of ERK, p38 or JNK signaling pathways in U-II-induced IL-8 expression, HUVECs were treated with a chemical inhibitor specific to each pathway and then stimulated with U-II. U0126, a dual mitogen-activated and extracellular regulated kinase kinase 1 (MEK1) and MEK2 inhibitor, and SB203580, a p38 inhibitor, were observed to be potent in the inhibition of U-II-stimulated IL-8 expression (Figure 3). Figure 3A shows that inhibition of ERK and p38 restrained U-II induction of IL-8 mRNA levels, indicating the involvement of ERK and p38 MAPK signaling pathways. In contrast, JNK inhibitor SP600125 alone had no significant effect on U-II induction of IL-8 mRNA. To determine the involvement of ERK, p38 or JNK signaling pathways in IL-8 protein secretion, UO126 and SB203580 were observed to be significantly potent in the inhibition of U-II induction of IL-8 protein secretion (Figure 3B).

### Increased transcriptional activity of the IL-8 promoter by U-II through NF-κB and AP-1 pathways

**Figure 4:**
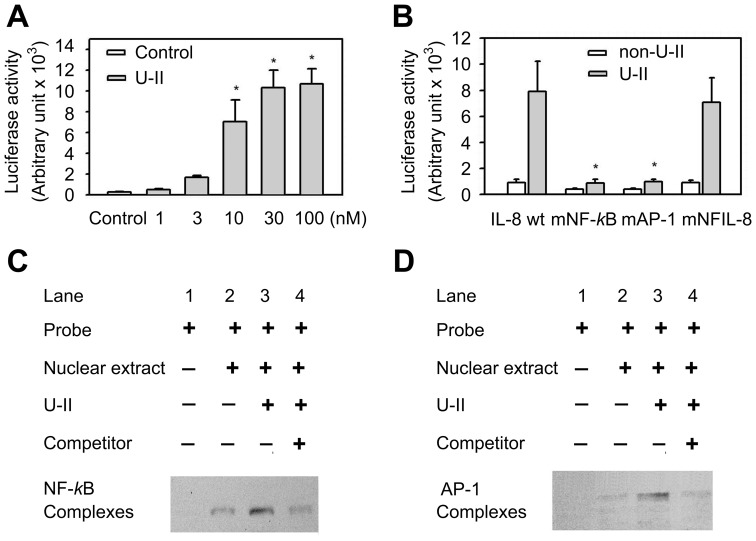
Transcriptional regulation of IL-8 expression by U-II. (A) Induction of IL-8 promoter activity by different concentrations of U-II. HUVECs were stimulated with U-II for an additional 24 h and then subjected to the luciferase assay of the wild-type IL-8 promoter construction. Data are expressed as the mean ± S.E.M. (*n* = 6). *P<0.05, compared with vehicle-treated control. (B) Luciferase assay of site-directed mutagenesis of the IL-8 promoter. HUVECs were transiently transfected with different IL-8 promoter plasmids for 24 h. Cells were then treated with U-II (30 nM) for 24 h. The mutant IL-8-luciferase constructs: IL-8 promoter plasmids, -*133IL-8*, wild-type IL-8 promoter; IL-8 nuclear factor (NF)-*κ*B mutation promoter plasmids, *mNF-κB*, NF-κB mutation; IL-8 AP-1 mutation promoter plasmids, *mAP-1*, AP-1 mutation; and IL-8 NFIL-6 mutation promoter plasmids, *mNF-IL6*, NF-IL-6 mutation. Data are expressed as the mean ± S.E.M. (n = 6). *P<0.05, compared with wild-type. The U-II–stimulated NF-*κ*B-binding capability (C), and AP-1-binding capability (D) in HUVECs. This binding activity was measured by using the electrophoretic mobility shift assay. Cells were incubated with with 30 nM U-II for 6 h. *Competitor* denotes a 200-fold molar excess of unlabeled oligonucleotide relative to the labeled probe; this was added to the binding assay for competition with the unlabeled oligonucleotide. A representative of three experiments as shown.

The luciferase reporter assay of the wild-type IL-8 promoter also shows that treatment with U-II increased the transcriptional activity of the IL-8 promoter as compared with no U-II treatment. No luciferase activity was observed in the mock-transfected and non-transfected control cells (Figure 4A). In order to better establish the contribution of individual *cis* elements of the IL-8 promoter in conferring responsiveness to U-II treatment, three mutant constructs, including AP-1, NF-IL-6, and NF-*κ*B, were subjected to the reporter assay, (Figure 4B). These mutations correspond to sequences known to disrupt the binding of the relevant transcription factors. The site-directed mutagenesis indicated that the mutation of AP-1 and NF-*κ*B binding sites reduced the promoter activities compared with those in the wild-type construct (Figure 4B). There was no significant difference between the NF-IL-6 mutant and the wild type. These results suggest the potential role of the elements, AP-1 and NF-*k*B, in IL-8 gene transcription following U-II treatment. As NF-κB and AP-1 transcription factors play key roles in the induction of IL-8 expression, the effects of U-II on NF-κB and AP-1 DNA binding activities were examined. U-II-mediated activation of NF-κB and AP-1 in the IL-8 promoter was studied with electrophoretic mobility shift assay. As shown in Figure 4C, nuclear extracts from control cells showed weak binding to the NF-κB probe (*lane 2*). In contrast, the binding capacity was significantly higher in cells stimulated with U-II (*lane 3*); this effect could be competed away with a 200-fold excess of the unlabeled NF-κB probe (*lane 4*). Similar results were obtained in the experiments using the AP-1 probe (Figure 4D). This experiment showed strong stimulation of IL-8 gene activation with U-II, in which the presence of multiple elements was required for full activation of the IL-8 promoter.

### The role of IL-8 RNA interference in the proliferation of HUVECs induced by U-II

**Figure 5:**
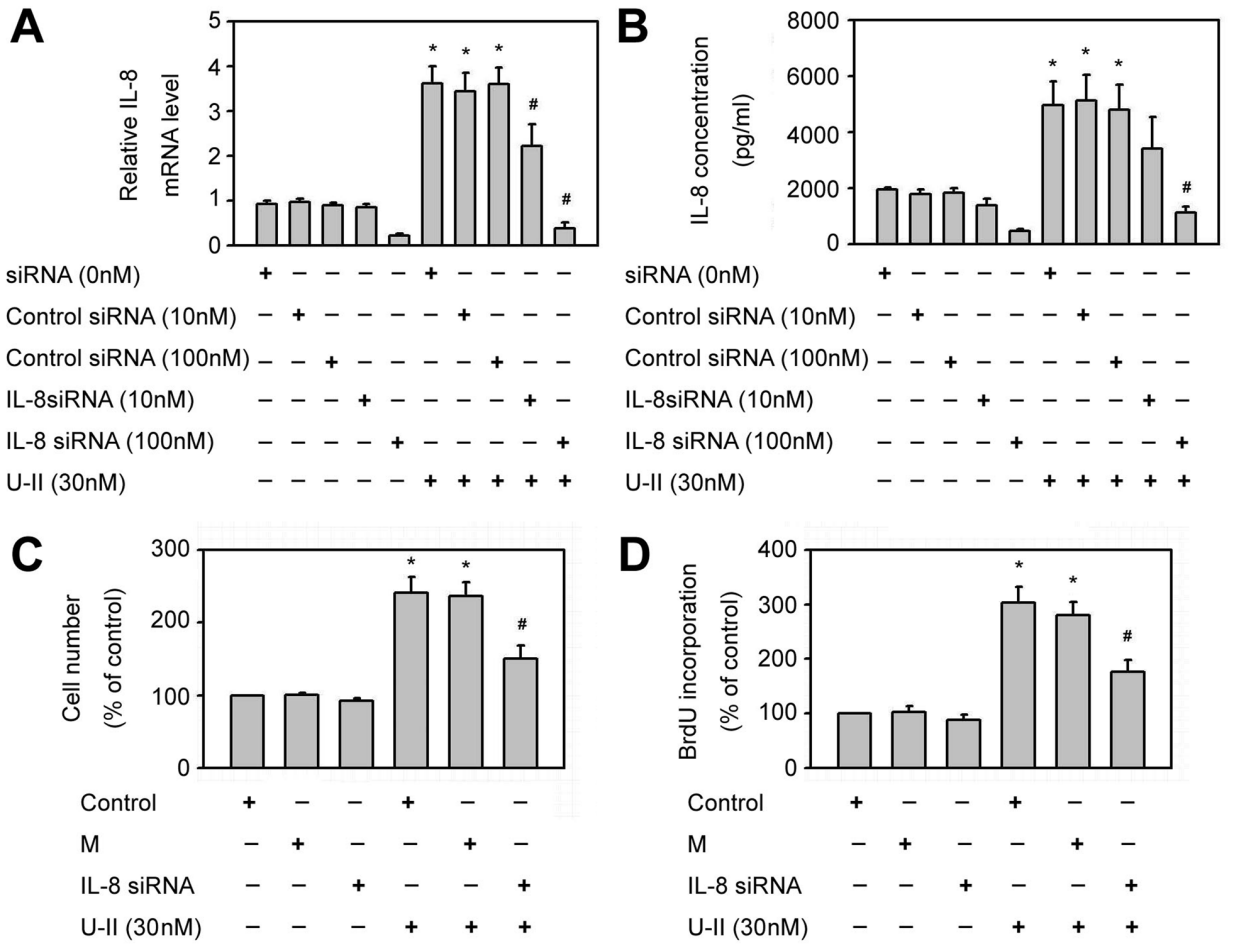
Effects of siRNA of IL-8 on HUVECs proliferation induced by U-II. *P<0.05 vs. control siRNA; #*P*<0.05 vs. control siRNA with U-II treatment. (A) The effect of IL-8 siRNA transfection on IL-8 mRNA levels in HUVECs. The cells were either transfected with control siRNA (10 or 100 nM) or transfected with IL-8 siRNA (10 or 100 nM) for 24 h to get IL-8 knockdown cells, and then treated 30 nM U-II for 24 h. Results were shown in mean±S.E.M. (*n* = 5). (B) The effect of IL-8 siRNA transfection on IL-8 protein expression in supernatants of HUVECs. The cells were either transfected with control siRNA (10 or 100 nM) or transfected with IL-8 siRNA (10 or 100 nM) for 24 h to get IL-8 knockdown cells, and then treated 30 nM U-II for 24 h. Results were shown in mean±S.E.M. (*n* = 5). (C) U II-increased cell proliferation was attenuated by IL-8 siRNA transfection in HUVECs. The cells were either transfected with control siRNA (100 nM) as mock controls (M) or transfected with IL-8 siRNA (100 nM) for 24 h to get IL-8 knockdown cells. Cells were then incubated with U-II (30 nM) for 24 h and then counted for cell number. Cell number was expressed as percentage of control. Results were shown in mean±S.E.M. (*n* = 6). (D) U-II-increased DNA synthesis was attenuated by IL-8 siRNA transfection in HUVECs. The cells were either transfected with control siRNA (100 nM) as mock controls (M) or transfected with IL-8 siRNA (100 nM) for 24 h to get IL-8 knockdown cells. Cells were then incubated with U-II (30 nM) for 24 h. DNA synthesis was measured by using BrdU incorporation and calculated as a percentage of the control value. Results were shown in mean±S.E.M. (*n* = 6).

To further determine the effects of U-II on IL-8 expression in endothelial cells, IL-8 siRNA was used for IL-8 knockdown in HUVECs. IL-8 siRNA significantly reduced IL-8 mRNA and protein expression in HUVECs compared with that in mock controls (Figure 5A and 5B). Compared with control treatment, U-II (30 nM) significantly enhanced both number of cells (Figure 5C) and DNA synthesis (Figure 5D) in HUVECs. U-II-increased cell proliferation was significantly inhibited by IL-8 RNA interference but not in mock controls as compared with U-II treatment alone (Figure 5C and 5D). These results support the role of IL-8 in U-II-induced cell proliferation in HUVECs.

## Discussion

Recent studies suggest that both U-II [7] and IL-8 play roles in vascular angiogenesis. This study examined the effects of U-II on IL-8 expression in HUVECs and the role of IL-8 in U-II-induced proliferation of HUVECs. U-II significantly increased both IL-8 mRNA and protein production in HUVECs in a time- and concentration-dependent manner. Moreover, [Orn^5^]-URP, U0126, and SB203580, inhibitors or blockers of UT, ERK and p38, respectively, could inhibit the increase in IL-8 mRNA and protein expression induced by U-II, so activation of these pathways might be involved in the U-II-induced IL-8 biosynthesis. Furthermore, U-II could induce proliferation of HUVECs. This effect could be inhibited by IL-8 siRNA, which indicates that IL-8 plays an important role in proliferation of HUVECs induced by U-II.

The mechanisms involved in the effect of U-II on IL-8 expression are similar to those on the induction of ET-1, TGF-β1 and cell adhesion molecules. U-II is able to stimulate TGF-β1 secretion from neonatal cardiac fibroblasts [22] and proximal tubular epithelial cells in rats [23] via UT activation. The mechanisms involved in the U-II-induced IL-8 expression are analogous to those involved in the U-II effects on cell proliferation. As far as the downstream signaling pathways are concerned, the involvement of MAPK as a result of UT stimulation is a common finding in the available literature, suggesting that this pathway plays a key role in the structure-modifying effects mediated by U-II. It has previously been suggested that U-II may stimulate the proliferation of vascular smooth muscle cells through MAPK signal transduction pathways [12]. U-II could induce IL-8 expression via the UT, MAPK, and p38 kinase pathways, which provided insights into U-II actions in endothelial cells. These signal pathways may play important roles in the activation of endothelial cells induced by U-II.

Previous reports have shown that AP-1 and NF-κB binding elements located in the IL-8 promoter region are essential for the transcriptional regulation of IL-8 gene expression in most cells [24]. Results of both reporter assay and electrophoretic mobility shift assay indicated that the transcription factors, AP-1 and NF-*κ*B, are involved in the U-II-induced IL-8 expression in HUVECs. MAPKs regulate IL-8 expression and secretion in a variety of cells including endothelial cells [25]. Previous studies showed that U-II activated ERK, p38 and JNK phosphorylation in HUVECs. However, pharmacological inhibitors of ERK and p38 but not JNK MAPKs inhibited U-II induction of IL-8 expression, indicating that ERK and p38 signaling pathways are required for U-II induction. U-II increased AP-1 DNA and NF-*κ*B binding activity, suggesting the role for AP-1 and NF-*κ*B in the induction of IL-8 expression. It remains to be determined if ERK and p38 MAPK pathways control AP-1 and/or NF-*κ*B DNA binding activity to increase IL-8 expression in HUVECs.

Strong evidence indicates the crosstalk between U-II and other vascular regulators plays an important role in the regulation of cell proliferation [12]. U-II could induce ET-1 expression in rat aortic smooth muscle cells via epidermal growth factor receptor transactivation [12]. Moreover, cell proliferation was increased in neonatal cardiac fibroblasts by U-II [20]. Other important angiogenic molecule, such as vascular endothelial growth factor (VEGF), has also been shown to be modulated by U-II [26], [27]. However, Albertin *et al* found that U-II did not affect VEGF expression in human endothelial cells [28]. The role of VEGF in U-II-induced IL-8 expression and even HUVEC proliferation warrant further study. The present study found that U-II could induce the expression of IL-8, an important factor in endothelial cell proliferation and vascular angiogenesis, and IL-8 siRNA could inhibit the effects of U-II on proliferation of endothelial cells, so IL-8 is involved in the U-II-induced proliferation of endothelial cells. In addition, the crosstalk between U-II and IL-8 in vascular regulation and vascular angiogenesis warrants further study.

In conclusion, U-II significantly increased both the mRNA and protein expression of IL-8 in HUVECs, via the activation of the UT, and the MAPK signal transduction pathways, and multiple promoter elements are involved in the IL-8 promoter activation during U-II stimulation. In addition, UII-induced proliferation of HUVECs could be inhibited by IL-8 siRNA. This study provides new insights into U-II actions that U-II may act partly via the autocrine production of IL-8 in HUVECs. Simultaneous blockade of U-II and of IL-8 expression could be a promising therapeutic strategy to moderate vascular angiogenesis.
