# Correction: Urotensin II Induces Interleukin 8 Expression in Human Umbilical Vein Endothelial Cells

**DOI:** 10.1371/journal.pone.0106275

**Published:** 2014-08-18

**Authors:** The PLOS ONE Staff

Affiliation 1 is incorrect. Please refer to the correct affiliation 1 and associated authors here:

Chung-Yi Lee, Yi-Tin Tsai

Department of Cardiovascular Surgery, Tri-Service General Hospital, National Defense Medical Center, Taipei, Taiwan, Republic of China.
